# On the Special Requirements of Ophthalmic Hospitals

**Published:** 1892-05-14

**Authors:** 


### ON THE SPECIAL REQUIREMENTS OF OPHTHALMIC HOSPITALS.

Hospitals for the treatment of diseases of the eye require arrangements of a somewhat different type to those proper to a general hospital. In the first place, the great bulk of the work is done, not in the wards, but in the out-patient department, the size of which must bear a very much larger relative proportion to the in-patient department than in other hospitals. Then, of the out-patients themselves, a very large proportion are either wholly or partially blind, and need every help that can be given them to guide them safely from one place to another. It is needless to say that staircases should be avoided if possible, but the exigencies of the site may not always allow this. In cases, then, where the patients must be taken up or down stairs, handrails should be placed on each side of the staircase, and the width of the steps should not be so great as to prevent a patient from holding each handrail with comfort. So also with passages, none of which should be so wide that a patient could not easily guide himself along if necessary, by feeling his way by both sides.

Another important point to consider carefully is the way in which doors open and shut. In the first place, exit doors must never be used for entrance, or *vice versa*. Then all doors must open in the way the patients go—that is to say, that if a particular door is to be used for entrance to a room, it must open into that room, and conversely the door by which patients leave the room must open out of it. This rule will involve no little care and forethought in fixing the positions of doorways, but, if strictly adhered to, it will prevent the possibility of patients running tilt at the edge of an open door, and possibly injuring an eye beyond repair.

In setting out the plan of the out-patient department, care must, of course, be taken that the patient does not anywhere retrace his steps, or cross or come into contact with a stream of patients going in a contrary direction. This precaution, though applicable to all hospitals, is doubly necessary in dealing with a crowd of blind or semi-blind people.

A spectacle-room is also a necessary adjunct to the out-patient department, and should be placed close to the dispensary and the exit.

For in-patients, the same careful precautions with regard to doors and, indeed, to all salient angles, are required. Beyond this, there is nothing of any special character required beyond what is necessary in an ordinary hospital.

